# Autocrine androgen action is essential for Leydig cell maturation and function, and protects against late-onset Leydig cell apoptosis in both mice and men

**DOI:** 10.1096/fj.14-255729

**Published:** 2014-11-17

**Authors:** Laura O’Hara, Kerry McInnes, Ioannis Simitsidellis, Stephanie Morgan, Nina Atanassova, Jolanta Slowikowska-Hilczer, Krzysztof Kula, Maria Szarras-Czapnik, Laura Milne, Rod T. Mitchell, Lee B. Smith

**Affiliations:** *MRC Centre for Reproductive Health and ^†^BHF Centre for Cardiovascular Science, University of Edinburgh, The Queen’s Medical Research Institute, Edinburgh, United Kingdom; ^‡^Institute of Experimental Morphology and Anthropology with Museum, Bulgarian Academy of Sciences, Sofia, Bulgaria; ^§^Department of Andrology and Reproductive Endocrinology, Medical University of Lodz, Lodz, Poland; and ^¶^Clinic of Endocrinology and Diabetology, Children's Memorial Health Institute, Warsaw, Poland

**Keywords:** testis, testosterone, estrogen, steroidogenesis, spermatogenesis

## Abstract

Leydig cell number and function decline as men age, and low testosterone is associated with all “Western” cardio-metabolic disorders. However, whether perturbed androgen action within the adult Leydig cell lineage predisposes individuals to this late-onset degeneration remains unknown. To address this, we generated a novel mouse model in which androgen receptor (AR) is ablated from ∼75% of adult Leydig stem cell/cell progenitors, from fetal life onward (Leydig cell AR knockout mice), permitting interrogation of the specific roles of autocrine Leydig cell AR signaling through comparison to adjacent AR-retaining Leydig cells, testes from littermate controls, and to human testes, including from patients with complete androgen insensitivity syndrome (CAIS). This revealed that autocrine AR signaling is dispensable for the attainment of final Leydig cell number but is essential for Leydig cell maturation and regulation of steroidogenic enzymes in adulthood. Furthermore, these studies reveal that autocrine AR signaling in Leydig cells protects against late-onset degeneration of the seminiferous epithelium in mice and inhibits Leydig cell apoptosis in both adult mice and patients with CAIS, possibly via opposing aberrant estrogen signaling. We conclude that autocrine androgen action within Leydig cells is essential for the lifelong support of spermatogenesis and the development and lifelong health of Leydig cells.—O’Hara, L., McInnes, K., Simitsidellis, I., Morgan, S., Atanassova, N., Slowikowska-Hilczer, J., Kula, K., Szarras-Czapnik, M., Milne, L., Mitchell, R. T., Smith, L. B. Autocrine androgen action is essential for Leydig cell maturation and function, and protects against late-onset Leydig cell apoptosis in both mice and men.

Male health and well-being are androgen dependent. Reduced androgen action is associated with many increasingly prevalent chronic and age-related clinical conditions, including cardiovascular disease (1), diabetes, obesity, and metabolic syndrome (2–5), and is a predictor of early death (6, 7). The testicular Leydig cells produce the vast majority of androgens in men and, as such, are a key driver of men’s health. Once established, the adult Leydig cells are maintained as a stable, terminally differentiated population with little evidence of cell turnover throughout the majority of adulthood (8). However, men experience both a reduction in Leydig cell number (9, 10) and reduced testosterone (T) production per Leydig cell (11) as they age, but the underlying cause for this is unclear.

The trophic functions of androgens are largely mediated throughout the body by binding the androgen receptor (AR), a member of the nuclear receptor superfamily of ligand-activated transcription factors. Fetal Leydig cells do not express AR (12); however, adult Leydig cells acquire AR expression and thus become responsive to autocrine androgen signaling at their stem/progenitor cell stage (in fetal life) (13). Why autocrine androgen signaling is required in stem/progenitor adult Leydig cells is unknown, but the presence of AR in these cells in fetal life could provide a mechanistic explanation as to why aberrant androgen action in fetal life predicts lower T levels and reduced androgen action in adulthood (14, 15). Indeed, recent data from our group support the notion that androgenic programming of adult Leydig stem cell/cell progenitors may have lifelong consequences (16).

Leydig cell androgen signaling has been postulated to be essential for the development of fully functional adult Leydig cells, specifically controlling their final number, their steroidogenic enzyme expression, and the maturation stage they reach (17). Hypotheses concerning the autocrine role of AR in Leydig cells have largely been formulated through studies using an AR null testicular feminization mouse (*Tfm*) (18). Leydig cell number in the *Tfm* is 60% controls (19), associated with a significant reduction in T production despite high levels of circulating LH (20, 21), which implies that steroidogenic enzyme expression is also altered. Indeed, transcript levels of several steroidogenic enzymes are almost absent in the *Tfm* testis (19). These changes in transcription are supported by observations of CYP17A1 and HSD17B enzyme activity, which are also both markedly reduced in the *Tfm* testis (20, 22). Several lines of evidence arising from the *Tfm* studies point to the importance of Leydig cell AR signaling in the maturation of Leydig cells to the adult Leydig cell stage. Gene expression of specific transcripts associated with fully mature adult Leydig cells, including *Insl3*, *Hsd3b6*, and *Ptgds*, is absent in *Tfm* testes (19). Furthermore, Leydig cells in the *Tfm* display prominent lipid droplets that are characteristic of immature adult Leydig cells, and the increase in smooth endoplasmic reticulum associated with normal Leydig cell maturation is absent (22).

However, the use of the *Tfm* model to delineate the role of AR in Leydig cells is complicated both by the effects of the absence of AR in other cells in the testis and the hypothalamic-pituitary-gonadal axis, and also the impacts of cryptorchidism associated with the *Tfm* mutant, which leads to temperature-induced degenerative effects (23). Conditional gene targeting has provided novel insights into the impact of AR signaling in multiple cell types within the male reproductive system (24–31) by circumventing the compounding effects associated with global ablation of AR function seen in the *Tfm* (32). A previous attempt to create a Leydig cell androgen receptor knockout (LCARKO) mouse using the Cre-*LoxP* system utilized AMHR2-Cre to drive AR ablation (33). These mice demonstrated a reduction in T secretion and testicular size and spermatogenic arrest at the round spermatid stage leading to infertility. However, because AMHR2-Cre also functions in Sertoli cells (34), the contribution of AR ablation in Sertoli cells to the overall phenotype makes it challenging to dissect the role that Leydig cell AR plays in this phenotype.

To establish the role of AR in the adult Leydig cell lineage, we generated a novel mouse model in which AR is ablated from ∼75% of adult Leydig stem cell/cell progenitors, from fetal life onward (LCARKO mice), permitting interrogation of the specific roles of autocrine Leydig cell AR signaling through comparison to adjacent AR-retaining Leydig cells, testes from littermate controls, and to normal human testes and those from patients with complete androgen insensitivity syndrome (CAIS), arising through mutation of AR. These analyses both confirm and refute some of the previously ascribed roles to AR in adult Leydig cells and reveal a previously unattributed role for autocrine AR signaling within developing Leydig cells essential for retention of the Leydig cell population in adulthood in both mice and humans. We conclude that autocrine androgen action during Leydig cell development is essential for the lifelong support of spermatogenesis and health of the Leydig cell population in adult males.

## MATERIALS AND METHODS

### Ethics statement

The ethics approval for human testicular biopsies was obtained from the Bioethics Committee at the Medical University of Lodz, Poland (reference number RNN/28/10/KE). All mice were bred under standard conditions of care and use under licensed approval from the UK Home Office (60/4200).

### Lineage tracing of adult Leydig stem/progenitor cells

Male congenic C57BL/6J mice hemizygous for a Fatty Acid Binding Protein 4 (*Fabp4*)-Cre transgene (35) were mated to homozygous R26R-EYFP females (36). The +/*Fabp4*-Cre;+/R26R-EYFP and ^+/+^;+/R26R-EYFP (control) male offspring from these matings were genotyped as previously described (30). Freshly dissected organs were visualized with a Leica MZFLIII microscope (Wetzlar, Germany) and an epifluorescent yellow fluorescent protein (YFP) filter. Photographs were taken with a CoolSNAP camera and PMCapture Pro 6.0 software (Photometrics, Tucson, AZ, USA).

### Targeted ablation of AR from Leydig cells using *Fabp4*-Cre mice

Mice in which AR has been ablated selectively from Leydig cells were generated using Cre/*loxP* technology. Male congenic C57BL/6J mice carrying a random insertion of *Fabp4*-Cre (35) were mated to C57BL/6J female mice homozygous for a floxed AR (24). The +/Cre;AR^flox^/y male offspring from these matings were termed “LCARKO,” whereas the ^+/+^;AR^flox^/y littermates were used as controls, termed “control.” +/Cre mice were also generated by mating *Fabp4*-Cre stud males to C57BL/6J females to confirm that expression of Cre alone did not induce a phenotype. Mice in which AR has been selectively ablated from adipocytes were generated by mating male congenic C57BL/6J mice hemizygous for a targeted insertion of Cre recombinase downstream of the adiponectin start codon (37) to homozygous female AR^flox^ mice. The +/Cre;AR^flox^/y male offspring from these matings were termed “Adipo-ARKO.” Mice in which AR has been ablated from all cells were generated by mating male congenic 129S1/SvImJ mice hemizygous for a targeted insertion of Cre recombinase at the ubiquitously expressed hypoxanthine phosphoribosyltransferase locus (38) to homozygous female AR^flox^ mice, as described previously (39). The +/Cre;AR^flox^/y male offspring from these matings were termed “ARKO.” Sex and genotype ratios were all identified at the expected Mendelian ratios.

### PCR genotyping of mice

Mice were genotyped for the inheritance of Cre recombinase as previously described (30). PCR amplification products were resolved using a QIAxcel capillary system (QIAGEN, Crawley, United Kingdom). An amplicon of 324 bp acted as a positive internal control, whereas an amplicon of 102 bp indicated the inheritance of the Cre recombinase transgene.

### Recovery of reproductive tissues

Mice were culled by inhalation of carbon dioxide and subsequent cervical dislocation (adults) or decapitation (neonates). If human chorionic gonadotropin (hCG) stimulation of Leydig cells was required, mice were injected s.c. with a single 20 IU dose of hCG (Pregnyl; Organon, Oss, The Netherlands), then culled 16 h later. Body weight and anogenital distance were measured, and mice were examined for any gross abnormalities of the reproductive system. Testes and seminal vesicles were removed and weighed. Tissues were fixed in Bouin’s fixative (Clin-Tech, Guildford, United Kingdom) for 6 h. Bouin-fixed tissues were processed and embedded in paraffin wax, and 5 *µ*m sections were used for histologic analysis as reported previously (30). Sections of testis were stained with hematoxylin and eosin using standard protocols and examined for histologic abnormalities.

### Immunohistochemistry

Immunolocalization was performed by a single-antibody colorimetric 3,3′-diaminobenzidine (DAB) immunostaining method, as described previously (27), a single- or double-antibody tyramide fluorescent immunostaining method, as described previously (26, 40), or an automated Bond immunostaining method, as described previously (27, 40). Antibodies used are listed in **Table 1**. A minimum of 5 individual sections for each age and genotype were immunostained in each experiment.

**TABLE 1.** Immunohistochemistry performed in this study, listing antibody source and method used

Protein stained for	Method	Primary antibodies
HSD3B/YFP	Double fluorescence	HSD3B: Santa Cruz Biotechnology #sc30820
		YFP: Life Technologies #A-11122
HSD3B/AR	Double fluorescence	HSD3B: Santa Cruz Biotechnology #sc30820
		AR: Abcam #ab74272
HSD3B	DAB	Santa Cruz Biotechnology #sc30820
INSL3	Bond	Gift from Steven Hartung
HSD17B3	DAB	HSD17B3: Santa Cruz Biotechnology #sc-135044
HSD17B3/AR	Double fluorescence	HSD17B3: Santa Cruz Biotechnology #sc-135044
		AR: Abcam #ab74272
CYP17A1/AR	Double fluorescence	CYP17A1: Santa Cruz Biotechnology #sc30820
		AR: Abcam #ab74272
CASP3	DAB	Cell Signaling (New England BioLabs) #96615
CASP3/AR	Bond	CASP3: Abcam #ab4051
		AR: Abcam #ab74272
ESR1	Single fluorescence	Santa Cruz Biotechnology #sc542
Pan-laminin	Bond	Abcam #ab11575
ACTA2	Bond	Sigma-Aldrich #A-2547
DES	Bond	Dako #M-0760
CDKN1B	DAB	Novacastra #NCL-p27

### Determination of testicular Leydig cell composition

Standard stereologic techniques involving point counting of cell nuclei were used to determine the nuclear volume per testis of each population of Leydig cells, namely normal adult Leydig cells positive for 3*β*-hydroxysteroid dehydrogenase (HSD3B), as described previously (24, 30). Data were used to determine the nuclear volume and number of Leydig cells per testis. Testes from a minimum of 4 individuals were counted for each group.

### Determination of testicular germ cell composition

Standard stereologic techniques involving point counting of cell nuclei were used to determine the nuclear volume per testis of spermatogonia, spermatocytes, and spermatids as described previously (24). Testes from a minimum of 4 individuals were counted for each group. Data were used to determine the nuclear volume per testis of each type of germ cell.

### Quantification of AR ablation in Leydig cells

For the quantification of AR ablation in Leydig cells of control and LCARKO mouse testes (*n* = 4 for each group), double-immunofluorescence detection was performed for AR and HSD3B as described above, and the sections were tiled using the Zeiss LSM 510 Meta Axiovert 100M confocal microscope and the Zen 2011 software (both from Carl Zeiss Limited, Welwyn Garden City, United Kingdom). The whole-testis images were then processed with the Image-Pro Plus 6.2 software (Media Cybernetics U.K., Berkshire, United Kingdom) to quantify AR ablation in Leydig cells. Cells demonstrating >50% HSD3B staining surrounding their nucleus were considered Leydig cells, and those with green-stained nuclei were counted as AR-positive Leydig cells, whereas those with blue-stained nuclei were considered to have AR ablation.

### Quantification of Leydig cell apoptosis

For the quantification of apoptosis in the Leydig cells of 80- to 100-d-old and 6- to 9-mo-old LCARKO mouse testes (n = 4 for each group), immunohistochemistry for cleaved caspase-3 (CASP3) (as described in Table 1) was performed. No Leydig cell-cleaved caspase staining could be seen in testis sections from control animals, so these were not quantified. Sections were analyzed using Image-Pro Plus 6.2 software with a Stereology 5.0 plug-in (Media Cybernetics U.K.) using the ×63 objective on a Leitz DMRB microscope (Wetzlar, Germany) fitted with a Prior Pro-Scan automatic stage (Prior Scientific Instruments Limited, Cambridge, United Kingdom). The Count (NV) setting was used to count all cells with Leydig cell morphology staining either positive or negative for cleaved caspase, and these figures were expressed as a percentage.

### Determination of genomic ablation of AR

DNA was isolated from a piece of frozen testis using a Mouse Tail Genomic DNA kit (Gen-Probe Life Sciences Limited, Manchester, United Kingdom) according to the manufacturer’s instructions and subjected to PCR amplification using primers 5′-GCTGATCATAGGCCTCTCTC-3′ and 5′-TGCCCTGAAAGCAGTCCTCT-3′ as described previously (26). PCR amplification products were resolved using a QIAxcel capillary system. An amplicon of 1142 bp indicated the presence of a floxed AR, whereas an amplicon of 612 bp indicated recombination between *loxP* sites and deletion of AR exon 2.

### Quantitative RT-PCR

RNA was isolated from frozen testes from 8, 80-d-old LCARKO and 8 control mice, or 6 Adipo-ARKO or 6 control mice, using the RNeasy Mini extraction kit with RNase-free DNase on the column digestion kit (QIAGEN) according to the manufacturer’s instructions. For quantitative RT-PCR, 5 ng Luciferase mRNA (Promega Corporation, Madison, WI, USA) was added to each testis sample before RNA extraction as an external standard (41). RNA was quantified using a NanoDrop 1000 spectrophotometer (Thermo Fisher Scientific, Waltham, MA, USA). Random hexamer-primed cDNA was prepared using the SuperScript VILO cDNA Synthesis Kit (Life Technologies, Grand Island, NY, USA) according to the manufacturer’s instructions. Quantitative PCR was performed on 80-d-old LCARKO and control testes for the genes listed in **Table 2**, using an ABI Prism 7900 Sequence Detection System (Applied Biosystems, Foster City, CA, USA) and the Roche Universal Probe library (Welwyn, United Kingdom), as described previously (30). The expression of each gene was related to an external positive control luciferase, as published previously (42), and all genes were expressed per testis.

**TABLE 2.** Quantitative RT-PCR assays used, with sequences of primers and UPL probe numbers for each assay

Transcript	5′ Primer	3′ Primer	Roche UPL probe
*Hsd3b6*	accatccttccacagttctagc	acagtgaccctggagatggt	95
*Ptgds*	ggctcctggacactacaccta	atagttggcctccaccactg	89
*Insl3*	aagaagccccatcatgacct	tttatttagactttttgggacacagg	10
*Cyp17a1*	catcccacacaaggctaaca	cagtgcccagagattgatga	67
*Hsd17b3*	aatatgtcacgatcggagctg	gaagggatccggttcagaat	5
*Hsd3b1*	gaactgcaggaggtcagagc	gcactgggcatccagaat	12
*Cyp11a1*	aagtatggccccatttacagg	tggggtccacgatgtaaact	104
*Cyp19a1*	gagagttcatgagagtctggatca	catggaacatgcttgaggact	55
*Sult1e1*	gaagaacaatccatccaccaat	tctgggaagtggttcttcca	6
*Esr1*	tgcaatgactatgcctctgg	ttgtagctggacacatgtagtcatt	93
*Gas6*	gcttctgctgctcctgct	gcctcctcgaagacttggta	27
*Pgr*	tgcacctgatctaatcctaaatga	ggtaaggcacagcgagtagaa	17

### Extraction of steroid hormones from testis and plasma

Immediately after culling, blood was collected from control and LCARKO mice by cardiac puncture with a syringe and needle pretreated with heparin. Plasma was separated by centrifugation and stored at −80°C. Testes were weighed, cut in half, and then snap-frozen on dry ice. Steroids were diethylether extracted from the testis lysate or plasma, dried under a constant stream of nitrogen, and then resuspended in the appropriate kit buffer.

### Quantification of hormone levels

T was measured using an ELISA kit (DEV9911; Demeditec Diagnostics, Kiel, Germany) according to the manufacturer’s instructions. Estradiol was measured using a mouse/rat estradiol ELISA kit (ES180S-100; Calbiotech, Spring Valley, CA, USA), which has been shown to provide good correlation to GC-MSMS-derived values (43) according to the manufacturer’s instructions. Progesterone was measured using a mouse progesterone ELISA kit (CSB-E05104m; Cusabio Biotech Company, Hubei Province, People’s Republic of China) according to the manufacturer’s instructions. LH and FSH were measured using in-house-designed ELISAs as previously described (44). All samples were run in a single assay for each hormone.

### Western blotting

Western blotting for CYP19A1 and ESR1 was performed as reported previously (45) on 80-d-old control and LCARKO testes. Blots were probed with primary antibodies ESR1 (sc-542; Santa Cruz Biotechnology, Dallas, TX, USA) and tyrosinated TUBA (*α*-tubulin) isoforms (ab6160; Abcam, Cambridge, MA, USA) or CYP19A1 [aromatase (Arom), ab18995; Abcam] and glyceraldehyde 3-phosphate dehydrogenase (GAPDH) (ab9486; Abcam). Primary antibodies were detected using either donkey anti-rabbit IRDye 680RD and goat anti-rat IRDye 800CW or donkey anti-goat IRDye 680RD and donkey anti-rabbit IRDye 800CW (all LI-COR Biosciences, Lincoln, NE, USA). Detection was carried out using the LI-COR Odyssey imaging system (LI-COR Biosciences) according to the manufacturer’s instructions.

### Bioinformatic investigation for evidence of direct AR regulation

Information about the genes *Hsd3b1*, *Hsd17b3*, and *Cyp11a1* and *mRNA* was obtained from the National Center for Biotechnology Information (http://www.ncbi.nlm.nih.gov/), and genomic DNA sequence was retrieved using the Evolutionary Conserved Regions’ browser (http://ecrbrowser.dcode.org/). The promoter and transcriptional start site for each gene was found using the Cold Spring Harbor Transcriptional Regulatory Element database (http://rulai.cshl.edu/cgi-bin/TRED/tred.cgi?process=home). Sequences, 3–7 kb upstream of the transcriptional start site and 3–5 kb 3′ of the translational stop codon, were then manually searched for putative androgen response element sequences and scored relative to the consensus sequence AGAACA/TnnnA/TGT/AA/GCT/A/C (weighting given to positions) [see (46, 47)].

### Statistical analysis

Data were analyzed using GraphPad Prism (version 5; GraphPad Software Incorporated, San Diego, CA, USA) using a 2-tailed unpaired *t* test (if comparing 2 groups), a 1-way ANOVA with Bonferroni *post hoc* test (if comparing >2 groups). Values are expressed as mean ± sem.

## RESULTS

### Identification of Leydig cell-specific Cre recombinase

**Figure 1.**
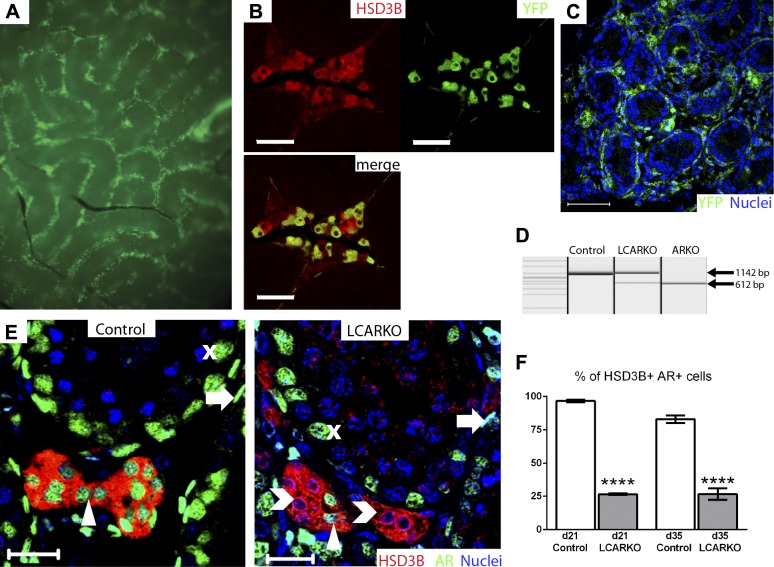
Localization and onset of *Fabp4*-Cre recombination. *A*) *Fabp4*-Cre activates YFP expression in the interstitial cells of an adult *Fabp4*-YFP testis. *B*) All YFP-expressing cells (green) in *Fabp4*-YFP testes are HSD3B-positive Leydig cells (red), but some Leydig cells do not express YFP. Scale bars, 50 *µ*m. *C*) YFP staining in d 0 *Fabp4*-YFP testis is present in a population of peritubular cells. Scale bar, 50 *µ*m. *D*) Genomic recombination of AR (indicated by a 612 bp band) occurs in a population of the cells of the d 0 LCARKO; another population of cells has unrecombined AR (1142 bp). Genomic AR in control testes is not recombined, and all genomic AR in ARKO testis is recombined. *E*) All HSD3B-positive cells (red) in d 21 control testis are positive for AR (green, arrowhead). Most HSD3B-positive cells in d 21 LCARKO testis are negative for AR (chevrons), but a small population are positive (arrowhead). Sertoli cells (X) and PTM cells (arrows) are positive for AR in both control and LCARKO. Scale bars, 20 *µ*m. *F*) When quantified, 97% of Leydig cells in d 21 control testis are positive for AR; this was significantly decreased in d 21 LCARKO testis (27%; ****P < 0.0001). Of Leydig cells in d 35 control testis, 83% are positive for AR; this is also significantly decreased in d 35 LCARKO testis (27%; ****P < 0.0001).

To permit targeting of Leydig cell AR, testes from several Cre recombinase transgenic mouse lines bred to a Cre-inducible YFP reporter gene [R26R-EYFP (36)] were screened for localization of testicular YFP expression (Supplemental Table S1). One line, in which Cre recombinase is driven by the promoter of *Fabp4* (previously known as *aP2*) (35), displayed Leydig cell-specific expression of YFP at d 75 (**Fig. 1*A*,** ***B***). Subsequent interrogation of *Fabp*4-Cre^+/−^;R26R-EYFP^+/−^ testes at d 0 revealed that the Cre recombinase first functions in the adult Leydig stem/progenitor cells, prior to the initiation of adult Leydig cell development (48) (Fig. 1*C*). Although fetal Leydig cells do not express AR (12), adult Leydig cells acquire AR expression at the stem/progenitor cell stage (13), and androgen signaling has been postulated to be essential for the development of fully functional adult Leydig cells, specifically controlling their final number, their steroidogenic enzyme expression, and the maturation stage they reach (17). We concluded that this line would permit specific ablation of AR from the adult Leydig cell population from the stem/progenitor cell stage onward.

### Generation of LCARKO mice

Testicular histology of hemizygous *Fabp4*-Cre males was normal when examined at d 365, confirming that the Cre recombinase transgene itself had no impact on testis function (Supplemental Fig. S1*A*). Male *Fabp4*-Cre^+/−^ mice were mated to female mice homozygous for a floxed allele of X-linked AR (AR^fl/fl^) (24) to generate LCARKO mice (*Fabp4*-Cre^+/−^;AR^fl/y^) and male littermates (*Fabp4*-Cre^−/−^;AR^fl/y^), which were used as controls throughout the study. Consistent with the ontogeny of *Fabp4*-Cre recombinase expression, LCARKO mice display evidence of AR DNA recombination within the testis when examined at d 0 (Fig. 1*D*). Subsequent immunohistochemical localization of AR protein confirmed that the loss of AR is restricted to the developing adult Leydig cell population (Fig. 1*E*); AR protein is retained in all other AR-expressing testicular cell types. Quantification of the number of developing adult Leydig cells expressing AR showed the loss of AR expression from approximately 75% of adult Leydig cells, which remained consistent when quantified at d 21 and 35 (Fig. 1*F*). Although initially thought to be a potentially confounding factor for downstream interpretation of results, the 25% of adult Leydig cells retaining AR protein meant that this model in fact provided a unique opportunity to determine the roles of autocrine AR signaling within Leydig cells *in vivo* because it permits comparison of adjacent Leydig cells, all exposed to the same endocrine and paracrine environment, with one difference: retention or absence of Leydig cell AR.

### LCARKO testes develop normally but display progressive testicular degeneration

**Figure 2.**
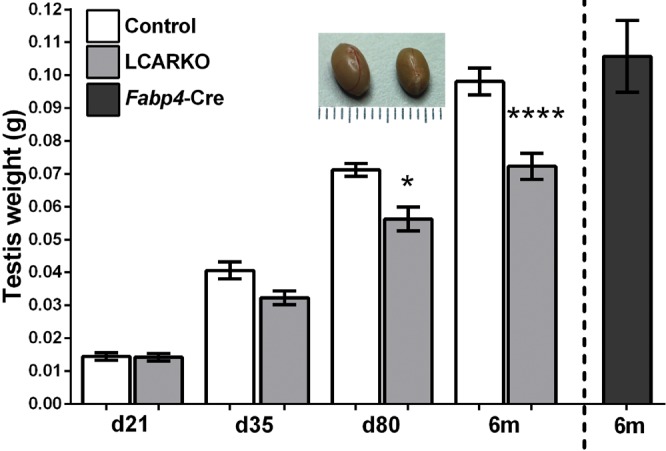
LCARKO mice have reduced testis size and weight. The mean weight of LCARKO testes is comparable with controls at d 21 (14.49 mg control; 14.21 mg LCARKO) and d 35 (40.62 mg control; 32.32 mg LCARKO). At d 80, the LCARKO mean testis weight is significantly reduced compared to controls (71.19 mg control; 56.28 mg LCARKO; *P < 0.05), and testes are visibly smaller (photo inset; scale bar increments of 1 mm). At 6 mo (6m) old, the mean LCARKO testes weight is also reduced compared to controls (98.08 mg control; 72.29 mg LCARKO; ****P < 0.0001). Error bars, sem.

**Figure 3.**
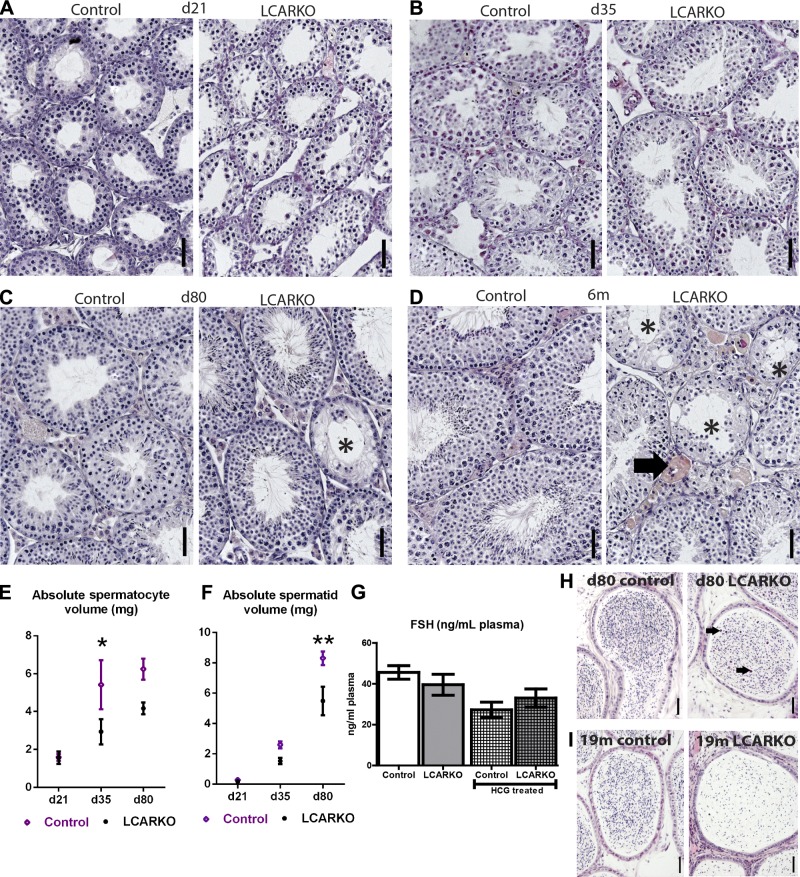
LCARKO mice have degenerative testicular histology. *A*) Testicular histology is similar between LCARKOs and controls at d 21. *B*) Testicular histology is similar between LCARKOs and controls at d 35. *C*) At d 80, LCARKO testes show foci of seminiferous epithelium degeneration (asterisk), whereas controls still appear normal. *D*) At 6 mo of age, foci of seminiferous epithelium degeneration are more widespread in the LCARKO (asterisks), which also have large pink/orange-staining multinucleate cells in the interstitium (arrow). Testes from controls are unaffected. *E*) LCARKOs have a reduction in testicular spermatocyte volume at d 35 (5.4 mg control; 3.0 mg LCARKO; *P < 0.05) and d 80 (6.3 mg control; 4.2 mg LCARKO) compared to controls. Error bars, sem. *F*) LCARKOs have a reduction in testicular spermatid volume compared to controls at d 35 (2.6 mg control; 1.5 mg LCARKO) and d 80 (8.3 mg control; 5.5 mg LCARKO; **P < 0.01). Error bars, sem. *G*) FSH levels are normal in LCARKO compared to controls at d 80 and for hCG treated at d 100. Error bars, sem. *H*) Spermatozoa are present in the cauda epididymis of both controls and LCARKOs at d 80, but immature round sloughed germ cells are also seen in the LCARKO (arrows). *I*) Fewer spermatozoa are present in the cauda of LCARKOs than controls at 19 mo. Scale bars, 50 *µ*m.

Having validated the generation of a LCARKO model, we next characterized the impact of the loss of Leydig cell AR on testicular architecture. Testes weight did not differ between LCARKO mice and control littermates when examined at d 21 or 35. However, a significant reduction in testis weight in LCARKO mice is observed at d 80 and at 6 mo (approximately d 180) (**Fig. 2**). Consistent with testis weight data, examination of testicular histology shows no apparent difference in testicular architecture between controls and LCARKO at d 21 and 35 (**Fig. 3*A, B***). CDKN1B staining is present in Sertoli cells in both control and LCARKO testes and ACTA2 (α-SMA) and pan-laminin staining in peritubular myoid (PTM) cells in both controls and LCARKO testes, demonstrating that these cells had matured appropriately (Supplemental Fig. S2). Although localization of the PTM intermediate filament protein desmin (DES) is interrupted in 80-d-old LCARKOs, suggesting that this may be an early effect of ablation of Leydig cell AR on PTM cell function. The first wave of spermatogenesis occurred normally, and all germ cell types are present. At d 80, testicular histology is normal in controls, but in the LCARKO, whereas much of the testis appears normal, localized seminiferous epithelium degeneration is observed at several foci throughout the testis (Fig. 3*C*). By 6 mo of age, seminiferous epithelium degeneration has become more widespread in LCARKO testes, whereas histology remains normal in 6-mo-old controls (Fig. 3*D*).

### Spermatogenesis is quantitatively reduced from d 35

Consistent with the late-onset epithelial degeneration, quantification of testicular cell numbers at d 21, 35, and 80 shows no significant difference in Sertoli or spermatogonial cell number (data not shown); however, a small but statistically significant reduction in spermatocyte (Fig. 3*E*) and spermatid (Fig. 3*F*) number is observed at d 35 and 80, suggesting that the ability of the testis to support quantitative, but not qualitative, spermatogenesis is perturbed at these ages. However, this is not reflected in circulating follicle-stimulating hormone (FSH) concentration, which remains unchanged between control and LCARKOs when examined at d 80 (Fig. 3*G*). The reduction in germ cell maturation is apparent upon examination of the epididymis. Mature spermatozoa can be seen in the lumen of the control cauda epididymis at d 80. In age-matched LCARKOs, a visible increase in the number of immature germ cells sloughed prematurely from the seminiferous epithelium is evident (Fig. 3*H*, arrows). Mature caudal spermatozoa are also present in 19-mo-old controls, but a visible reduction in the number of spermatozoa is apparent in age-matched LCARKO mice (Fig. 3*I*).

Similar to many Cre recombinase lines, *Fabp4*-Cre is expressed at varying levels in different tissues in the body (49) but has been primarily used to ablate AR in the CNS (50) and adipocytes (25), so it was necessary to rule out the possibility that the observed testicular phenotype resulted secondarily from the loss of AR in these tissues. Both androgen receptor knockout (ARKO) and brain-specific ARKO mice display changes in mating behavior (51); however, analysis of mating behavior in LCARKO mice identified no significant difference in the number of successful matings compared to controls (data not shown), demonstrating that any targeting of AR in the brain using Fabp4-Cre has minimal phenotypic impact. To rule out any influence due to the loss of AR from adipocytes, we exploited a second adipocyte-specific Cre recombinase line [adiponectin-Cre (*Adipoq*-Cre) (37)] to generate an adipocyte androgen receptor knockout (Adipo-ARKO) mouse. Although this line efficiently ablates AR from adipocytes, it did not ablate AR from any cells of the testis (Supplemental Fig. S1*B*). The resulting Adipo-ARKO mouse displays no testicular phenotype (Supplemental Fig. S1*C*), and gene expression of steroidogenic enzymes and Leydig cell maturation markers are unchanged (Supplemental Fig. S1*D*). We therefore concluded that the progressive testicular degeneration seen in LCARKO mice (Fig. 3) results directly from specific AR ablation from the adult Leydig cell population.

### Leydig cell AR signaling does not control final adult Leydig cell number

**Figure 4.**
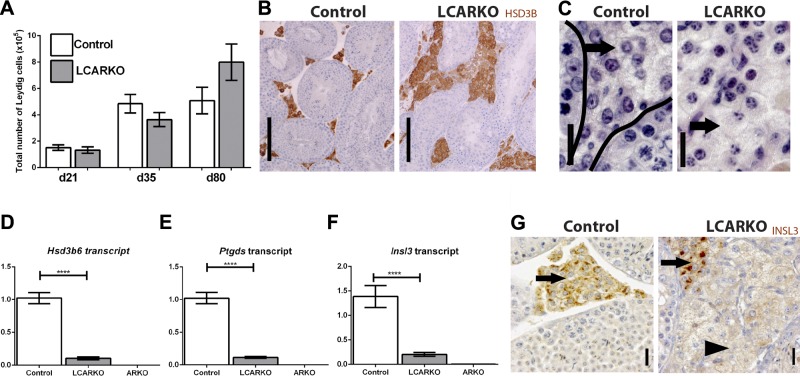
LCARKO Leydig cells reach final adult number but do not express mature Leydig cell markers. *A*) The total number of Leydig cells per testis is not significantly different between controls and LCARKOs at d 21, 35, and 80. Error bars, sem. *B*) The percentage of testis occupied by Leydig cells (staining for HSD3B) appears to be larger in the LCARKO than in the control. Scale bars, 100 *µ*m. *C*) The size of Leydig cell cytoplasm appears larger in LCARKOs than controls at d 80 (lines delineate seminiferous tubules in control panel; arrows indicate interstitium). Scale bars, 20 *µ*m. *D*) *Hsd3b6* is significantly decreased (****P < 0.0001) in LCARKO testis compared to controls at d 80 and is absent in ARKO testis. *E*) *Ptgds* is significantly decreased (****P < 0.0001) in LCARKO testis compared to controls at d 80 and is absent in ARKO testis. *F*) *Insl3* is significantly decreased (****P < 0.0001) in LCARKO testis compared to controls at d 80 and is absent in ARKO testis. *G*) INSL3 staining is widespread throughout the interstitium of control testes, but LCARKO testes show 2 populations of cells with Leydig cell-like morphology: a larger population that does not stain for INSL3 (arrowhead); and a smaller population that does (arrows). Scale bars, 20 *µ*m.

Having determined that the loss of Leydig cell AR induces a progressive degenerative phenotype in the testis, we next aimed to establish the specific role of AR in developing adult Leydig cells. Previously published literature states that AR functions in adult Leydig cells to determine final Leydig cell number (19). We quantified the number of Leydig cells at d 21, 35, and 80, by which age adult Leydig cells have fully matured and reached their final number. Surprisingly, Leydig cell number is not significantly different between control and LCARKO at any age examined (**Fig. 4*A***). Thus, in contrast to the published literature, these data suggest that autocrine AR action is not required within adult Leydig cells for the attainment of final adult Leydig cell number.

### Leydig cell AR signaling controls adult Leydig cell maturation

In addition to controlling Leydig cell number, AR in adult Leydig cells has been postulated to control adult Leydig cell maturation (19, 22). Ablation of AR from adult Leydig cells is associated with adult Leydig cell hypertrophy at d 80 (Fig. 4*B*) Leydig cell cytoplasm is increased in size, reminiscent of immature adult Leydig cells (Fig. 4*C*) (52). Consistent with this, expression of 3 genes previously demonstrated to be restricted to mature adult Leydig cells (*Hsd3b6*, *Ptgds*, and *Insl3*) is significantly reduced in LCARKO testes when examined at d 80, suggesting a failure of adult Leydig cell maturation (Fig. 4*D*–*F*). These gene expression data are further supported by immunohistochemical localization of the Leydig cell maturation marker INSL3, which is present in all Leydig cells of control testes but is restricted to a subset of Leydig cells within LCARKO testes (Fig. 4*G*). Together, these data strongly support the previously postulated role for Leydig cell AR in promoting adult Leydig cell maturation.

### Leydig cell AR signaling controls steroidogenesis

**Figure 5.**
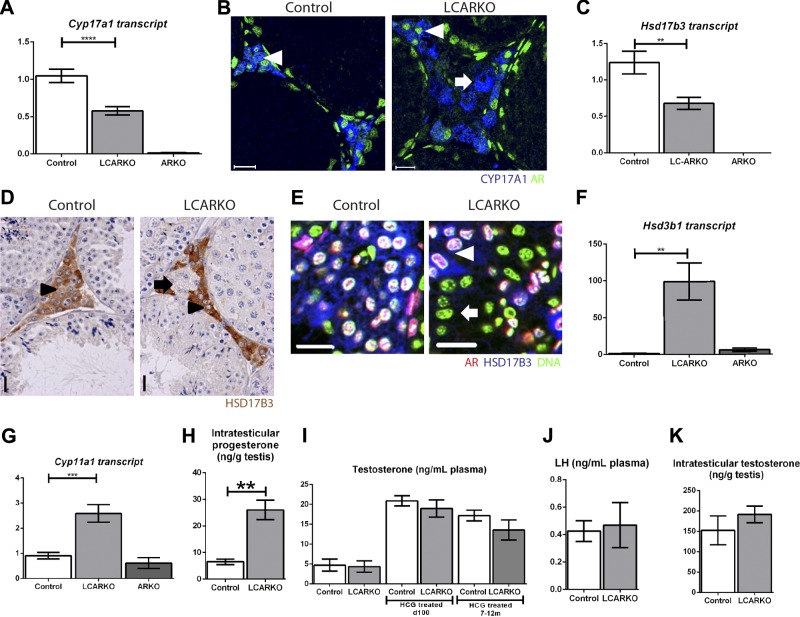
LCARKO testes have changes in steroidogenic enzyme expression but produce normal levels of T. *A*) *Cyp17a1* gene expression is significantly decreased (****P < 0.0001) in LCARKO testis compared to controls at d 80 and is absent in ARKO testis. *B*) AR immunostaining (green) is seen in the nuclei of CYP17A1 (blue)-positive Leydig cells in controls (arrowhead). Both AR-positive (arrowhead) and -negative (arrow) cells in LCARKOs express CYP17A1. Scale bars, 20 *µ*m. *C*) *Hsd17b3* gene expression is significantly decreased (**P < 0.01) in LCARKO testis compared to controls at d 80 and is absent in ARKO testis. *D*) Two populations of Leydig cells can be seen in the LCARKO: an HSD17B3-positive population (arrowheads), and an HSD17B3-negative population (arrow). Scale bars, 20 *μ*m *E*) AR immunostaining (red) is seen in the nuclei of HSD17B3 (blue)-positive Leydig cells in controls. In LCARKOs, HSD17B3 is localized to AR-positive cells (arrowhead), but not AR-negative cells (arrow). Scale bars, 20 *µ*m. *F*) *Hsbd3b1* gene expression is significantly increased (**P < 0.01) in LCARKO testis compared to controls at d 80. *G*) *Cyp11a1* gene expression is significantly increased (***P < 0.001) in LCARKO testis compared to controls at d 80. *H*) Intratesticular progesterone is significantly increased (**P < 0.01) in LCARKO compared to control mice at d 80. *I*) Plasma T levels are not significantly different between control and LCARKO mice at d 80, d 100 hCG treated, and 7–12 mo hCG treated. *J*) Plasma LH levels are not significantly different between control and LCARKO mice at d 80. *K*) ITT levels are not significantly different between control and LCARKO testes at d 80.

Previous data from the *Tfm* (ARKO) mouse model describe significant changes in expression levels and activity of steroidogenic enzymes, which have been attributed to the loss of AR in adult Leydig cells (19, 20, 22). To establish whether this is indeed a direct effect of the loss of AR signaling specifically from adult Leydig cells, we compared transcript and protein levels of these steroidogenic enzymes between LCARKO and complete-ARKO mice at d 80. mRNA expression of *Cyp17a1* is significantly reduced in LCARKO testes and is completely absent from ARKO testes, suggesting that Leydig cell AR influences expression of this enzyme (**Fig. 5*A***). However, double-immunofluorescence localization of AR and CYP17A1 in d 80 LCARKO testes reveals no obvious spatial correlation between the loss of AR and altered expression of CYP17A1 (Fig. 5*B*), suggesting that, whereas Leydig cell AR may influence gene expression, the complete absence of *Cyp17a1* expression observed in the ARKO model is not due to the loss of AR expression specifically from adult Leydig cells. In contrast, whereas *Hsd17b3* expression is also significantly reduced in LCARKO testes (Fig. 5*C*), immunohistochemistry for HSD17B3 in 80-d-old LCARKO testes (Fig. 5*D*) revealed that there are both HSD17B3-positive and HSD17B3-negative cell populations with Leydig cell morphology in the LCARKO testis. Double-immunofluorescence localization of AR and HSD17B3 in 80-d-old LCARKO testes (Fig. 5*E*) confirmed that whereas AR-positive Leydig cells in both controls and LCARKOs express HSD17B3, AR-negative Leydig cells in the LCARKO do not, suggesting that HSD17B3 protein expression is dependent on autocrine AR action within Leydig cells. Surprisingly, whereas transcript levels of *Hsd3b1* and *Cyp11a1* do not differ between ARKO and control testes, both are significantly increased in LCARKO testes (Fig. 5*F* and *G*), suggesting that androgens normally act to attenuate gene expression of these enzyme transcripts. To examine whether increased expression of these 2 transcripts was functionally significant, we determined intratesticular progesterone concentrations, which would be enhanced by increased CYP11A1 and HSD3B1 activities. Indeed, a significant increase in intratesticular progesterone was detected at d 80 (Fig. 5*H*), consistent with this hypothesis. Together, these data suggest that autocrine AR action is required to positively and negatively regulate expression (and by doing so, activity) of key enzymes, in support of normal steroidogenesis.

### Seminiferous tubule degeneration in LCARKOs is independent of T concentration

Having characterized the role that adult Leydig cell AR plays in adult Leydig cell development and function, we moved to establish the cause of the degenerative testicular phenotype observed in LCARKO mice. A rat model of Leydig cell ablation by ethane dimethane sulfonate (EDS) and supplementation with T has shown that T is necessary and sufficient to support spermatogenesis (53). By inference, this suggests that the only product produced by Leydig cells necessary for spermatogenesis is T. Given the impact that Leydig cell AR loss has on adult Leydig cell maturation and steroidogenic enzyme expression, including loss of HSD17B3 protein from many Leydig cells, we hypothesized that the seminiferous tubule degeneration observed in the LCARKO mouse results from a reduction in adult Leydig cell T production. However, no significant difference in circulating T is observed between LCARKO and control mice when examined at d 80 (when seminiferous tubule degeneration is already apparent), or when control and LCARKO mice are injected with a high-dose hCG stimulus at d 100 or at 7–12 mo of age (Fig. 5*I*). Moreover, blood levels of LH are unchanged from control values at d 80 (Fig. 5*J*). Intratesticular testosterone (ITT) was also measured and found to be unchanged in LCARKOs compared to controls at d 80 (Fig. 5*K*). Together, these data suggest that androgen production by adult Leydig cells not only promotes spermatogenesis but also acts *via* Leydig cell AR to prevent seminiferous tubule degeneration, perhaps by suppressing aberrant production of another factor by adult Leydig cells, which is detrimental to spermatogenesis. The most obvious candidate for this factor is the increase in progesterone production, and the possibility for increased progesterone signaling; however, expression of *Pgr* was undetectable in the testis at d 80 (data not shown), effectively ruling this out.

### Loss of AR leads to late-onset Leydig cell apoptosis in LCARKO mice and patients with CAIS

**Figure 6.**
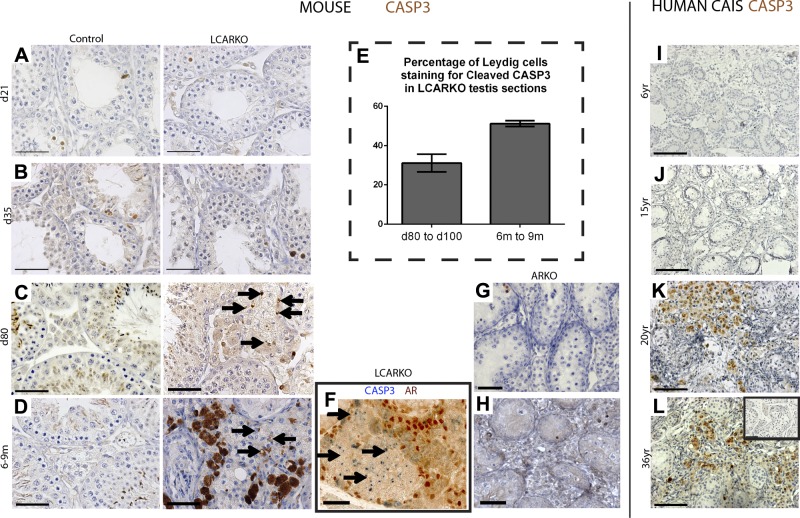
Apoptosis is seen in both aged LCARKOs and CAIS patient biopsies. *A* and *B*) No interstitial apoptosis (marked by CASP3 immunostaining) was observed in the interstitium of d 21, or d 35 control or LCARKO testes. A few germ cells were noted to stain for CASP3 in each section. *C*) No interstitial CASP3 immunostaining was observed in the interstitium of d 80 control mice. Punctate CASP3 immunostaining was observed in cells with Leydig cell morphology in d 80 LCARKO mice (arrows). *D*) Extensive interstitial apoptosis was noted in the interstitium of 6- to 9-mo-old LCARKO testes, unlike age-matched controls. *E*) When quantified, 31% of Leydig cells at d 80–100 and 51% of cells at 6–9 mo were found to immunostain positive for CASP3. *F*) CASP staining (blue) was noted in AR (brown)-negative cells (arrows), and not AR-positive cells.. *G* and *H*) No interstitial apoptosis was noted in either d 80 or aged ARKO mice. *I* and *J*) No apoptosis was noted in biopsies from 6- or 15-yr-old patients with CAIS. *K* and *L*) Extensive interstitial apoptosis was noted in biopsies from 20- and 36-yr-old patients with CAIS, whereas biopsies from normal adult men (inset) did not show apoptosis. Scale bars, 50 *µ*m.

To examine seminiferous tubule degradation in further detail, additional histologic inspection of LCARKO testes was undertaken. As a reduction in the second major adult Leydig cell product, INSL3 had been identified and that RXFPL, the receptor for INSL3, is expressed in germ cells (54), we hypothesized that impaired INSL3 signaling may result in germ cell apoptosis, which would explain the tubular degeneration. However, there is no visible difference in the number of apoptotic germ cells between the testes of LCARKOs and controls at d 21, 35, and 80 (**Fig. 6*A***–***D***), in agreement with a recent study suggesting that INSL3 is dispensable for spermatogenesis (55). However, surprisingly, given that the Leydig cell population normally displays minimal cell turnover (56), Leydig cell apoptosis is evident in the interstitium of the LCARKO testis at both d 80 and 100 and also at 6–9 mo of age (Fig. 6*C*, *D*). Staining at d 80–100 is punctate and in the cytoplasm of cells (Fig. 6*C*, arrows). By 6–9 mo, this punctate staining can still be seen (Fig. 6*D*, arrows), but large, completely stained cells can also be seen at this later age. Both of these staining patterns are classified as positive and quantified as 31% Leydig cells staining positive for cleaved CASP3 at d 80–100 and 51% at 6–9 mo (Fig. 6*E*). No Leydig cell apoptosis is noted in the interstitium of age-matched controls.

We hypothesized that the Leydig cell apoptosis could result from chronic hyperstimulation of Leydig cell steroidogenesis, which is known to increase cellular stress (57) in the ∼25% of Leydig cells retaining AR expression. These cells may be required to increase their T production to maintain circulating levels as a compensation for the poorly functioning AR knockout (KO) Leydig cells. However, hCG-mediated interrogation of maximal T production at either d 100 or 7–12 mo (Fig. 5*H*) reveals no significant difference between LCARKO and control animals, suggesting that Leydig cell production of T is unaffected by the loss of AR, and by inference, that the remaining AR-positive Leydig cells are not under obvious stress. In fact, contrary to this, double immunolocalization of CASP3 and AR shows that apoptosis was only observed in Leydig cells lacking AR expression. Adjacent Leydig cells retaining AR are unaffected (Fig. 6*F*). This is suggestive of a failure of an unknown AR-dependent autocrine function, which marks AR-negative Leydig cells for apoptosis. In contrast to the LCARKO mouse, Leydig cells in total-ARKO mice did not show signs of apoptosis at d 80 or 9 mo (Fig. 6*G*, *H*).

The LCARKO mouse model with its normal T levels and lack of functioning AR is reminiscent of CAIS in humans (normal/high T; no AR), unlike the *Tfm* or ARKO mouse, which has low T levels and absent steroidogenesis, and because we have recently identified that adult Leydig cells arise from the same stem cell population in both mice and humans (16), we assayed for apoptosis in testicular biopsies of patients with CAIS at different ages. Apoptosis is not detectable in younger patients aged 6 (Fig. 6*I*) or 15 (Fig. 6*J*) yr with CAIS, but patients aged 20 (Fig. 6*K*) and 36 (Fig. 6*L*) yr both show extensive interstitial cell apoptosis in cells with extensive cytoplasm that can be identified morphologically as Leydig cells. Biopsies from normal adult testes do not show Leydig cell apoptosis (Fig. 6*L*, inset).

### Increase in ESR1 signaling in LCARKO testis

**Figure 7.**
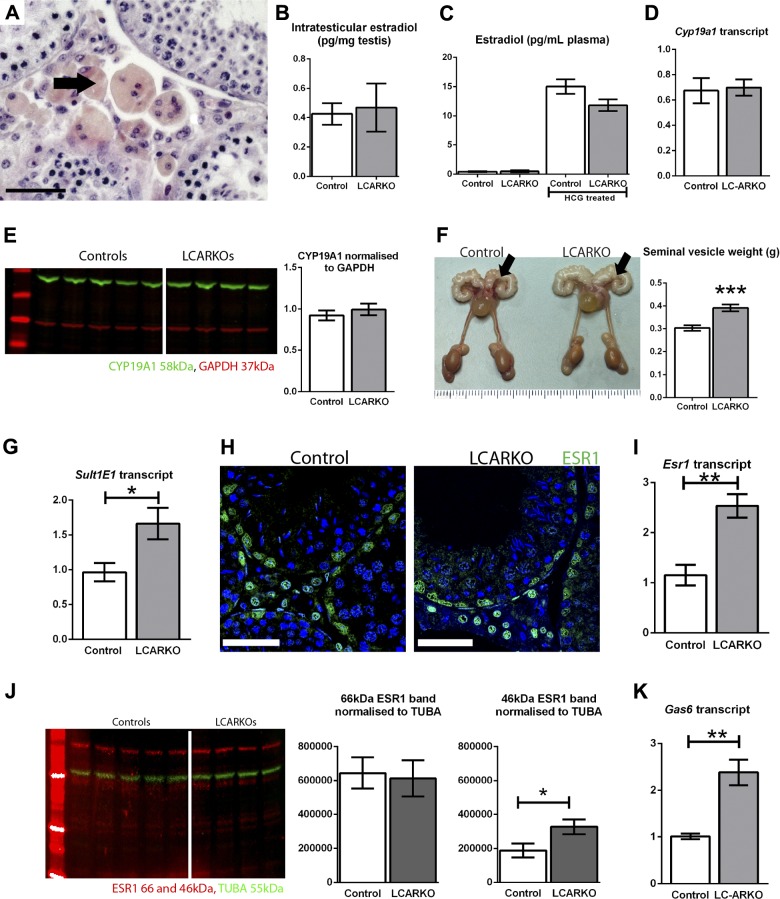
LCARKO testes show an increase in ESR1 signaling. *A*) Multinucleate cells that stain pink/orange with hematoxylin and eosin are noted in ≥6-mo-old LCARKO testis interstitium (arrow). Scale bar, 50 *µ*m. *B*) Intratesticular estradiol levels are not significantly different between control and LCARKO mice at d 80. *C*) Plasma estradiol levels are not significantly different between control and LCARKO mice at d 80 or for hCG treated at d 100. *D*) *Cyp19a1* is not significantly different in LCARKO testis compared to controls at d 80. *E*) Levels of CYP19A1 (green; 58 kDa) normalized to GAPDH (red; 37 kDa) are not significantly different between LCARKO and controls at d 100. Error bars, SEM. *F*) Seminal vesicles are visibly larger (arrows) and also significantly heavier (***P < 0.001) in LCARKO compared to control mice post 6 mo of age. *G*) *Sult1e1* gene expression is significantly increased (*P < 0.05) in LCARKO testis compared to controls at d 80. *H*) ESR1 (green) is localized to Sertoli, PTM, and Leydig cell nuclei in both control and LCARKO testis. Scale bars, 50 *µ*m. *I*) *Esr1* gene expression is significantly increased (**P < 0.01) in LCARKO testis compared to controls at d 80. *J*) Levels of the 46 kDa isoform of ESR1 (red) normalized to α-tubulin (red; 55 kDa) are significantly increased in LCARKO testes compared to controls at d 100 (*P* < 0.05), but the levels of the 66 kDa isoform are not significantly different. *K*) *Gas6* gene expression is significantly increased (**P < 0.01) in LCARKO testis compared to controls at d 80.

In addition to the evidence of Leydig cell apoptosis, phagocytic macrophages were noted in the testicular interstitium of adult LCARKO testes (**Fig. 7*A***, arrow). Two other mouse models described in the literature display a similar phenotype of progressive seminiferous tubule degeneration, both of which are associated with a chronic increase in estrogen signaling within the testis, either through over-expression of Arom (58) or KO of estrogen sulfotransferase (SULT1E1) (59). To assess the possibility that increased estrogen signaling is a mechanism underpinning our observations, we interrogated key components of the estrogen signaling system. We observed no significant difference in concentrations of either intratesticular or plasma estradiol between LCARKO and control animals (Fig. 7*B*, *C*), and the concentrations of *Cyp19a1* transcript (Fig. 7*D*) and CYP19A1 protein (Fig. 7*E*) are both unchanged in LCARKO testes compared to the control. Seminal vesicle weight is significantly increased in the LCARKO model, similar to that observed in the SULT1E1-KO (Fig. 7*F*); however, surprisingly, levels of the *Sult1e1* transcript are slightly increased (Fig. 7*G*).

We localized ESR1 (ER*α*) protein by immunohistochemistry using an antibody raised against the C-terminal of the mouse protein and found it to be present in the Leydig, PTM, and Sertoli cells of the testis in both control and LCARKO mice (Fig. 7*H*). A significant increase in *Esr1* transcript (Fig. 7*I*) and in the 46 kDa isoform of ESR1 protein (Fig. 7*J*) is noted in LCARKO compared to control testes. A significant increase in transcription of *Gas6*, a downstream target of ESR1 signaling in Leydig cells (60) (Fig. 7*K*), is also observed in the LCARKO testes, suggesting that the phenotypic changes associated with estrogen in the LCARKO may result from an increase in ESR1 signaling rather than an increased level of estradiol.

## DISCUSSION

To examine the role of androgen action within developing Leydig cells, we generated a novel mouse model lacking testicular AR specifically in the adult Leydig cell population, from the stem cell stage (17.5 d postconception) onward. Because 25% of Leydig cells retain AR, this model provides a unique opportunity to determine the roles of autocrine AR signaling within Leydig cells *in vivo* because it permits the comparison of adjacent Leydig cells, all exposed to the same endocrine and paracrine environment, with one difference: retention or absence of Leydig cell AR. We show that autocrine androgen action within developing adult Leydig cells is dispensable for the attainment of final Leydig cell numbers in adulthood but is essential for correct Leydig cell maturation and normal steroidogenic enzyme transcription. Although these defects do not initially impact testis function either in terms of spermatogenesis or T production, we show for the first time that failure of autocrine androgen action in developing Leydig cells eventually leads to seminiferous tubule degeneration in mice, and increased Leydig cell apoptosis in adulthood, possibly *via* a failure to oppose testicular estrogen signaling. Because a similar increase in Leydig cell apoptosis was observed in adult human patients with CAIS, in whom Leydig cell AR function is also absent, our findings may have relevance to humans.

### Autocrine AR signaling is dispensable for the attainment of normal Leydig cell numbers

Leydig cell number in the *Tfm* is 60% of controls (19), demonstrating that androgen signaling is essential for the attainment of final Leydig cell number. However, Leydig cell number in the LCARKO testis at d 21 and 35 and in adulthood at d 80 (before the onset of the degenerative phenotype) does not differ from control littermates. Furthermore, the proportion of AR-positive and AR-negative Leydig cells remains constant between d 21 and 35 (a time of intense Leydig cell proliferation), consistent with no difference in proliferation rate in Leydig cells that lack AR. Together, these data show that the loss of autocrine AR signaling within Leydig cells does not inhibit their proliferation or the attainment of final numbers. This suggests that the reduction in Leydig cells observed in the *Tfm* is due to the absence of androgen signaling in another cell type(s). Recent data from our group have demonstrated that Sertoli cells are key drivers of final Leydig cell numbers in adulthood (61), which is consistent with previous evidence suggesting that AR signaling within Sertoli cells may be the mechanism by which androgen signaling controls Leydig cell numbers (52, 62).

### Autocrine AR signaling is essential for Leydig cell maturation and normal steroidogenic enzyme transcription

Several lines of evidence arising from the *Tfm* studies point to the importance of Leydig cell AR signaling in the maturation of Leydig cells to the adult Leydig cell stage, including the absence of expression of the maturation markers *Insl3*, *Hsd3b6*, and *Ptgds* (19), retention of immature adult Leydig cell morphology, and a reduction in smooth endoplasmic reticulum normally associated with immature adult Leydig cells (22). Like the *Tfm*, maturation appears to be compromised in LCARKO Leydig cells. Loss of endogenous AR expression leads to a reduction in the adult Leydig cell-specific transcripts *Ptgds*, *Cyp17a1*, *Hsd17b3*, and *Hsd3b6*, as well as INSL3 at both the transcript and protein level. Conversely, whereas also absent in the *Tfm* testes, expression of the earlier-onset steroidogenic enzymes *Hsd3b1* and *Cyp11a1* is significantly increased in LCARKO testes compared to controls at d 80, leading to an increase in intratesticular progesterone. The reason for this is unclear, but this may reflect a compensatory mechanism for the loss of AR, a release from normal AR-mediated control, or a reflection of the arrested stage. Indeed, regulation of the genes *Hsd3b6*, *Ptgds*, *Insl3*, *Hsd17b3*, and *Cyp11a1* in response to androgen stimulation has been reported in a number of mouse or rat tissues and in transcriptomic studies in both rodent and human tissue and cells [identified *via* (63, 64)]. Despite the data summarized above, there is a paucity of information regarding the identification of sequences mediating AR binding and transcriptional regulation for the above genes. A notable exception is the Insl3 genes, where a region mediating androgen regulation has been mapped to sequences in the promoter, −132 to −85 (65). An empirical analysis of the genomic sequence 5′ and 3′ of the genes *Hsd3b1*, *Hsd17b3*, and *Cyp11a1*, as well as coding sequences, identifies a number of putative palindromic binding sites for the AR, with >80% identity with the consensus receptor binding sequence, together with a large number of half-sites matching the sequence AGAACA/T. The presence of these binding sites would be consistent with direct regulation of these genes by the AR (Supplemental Fig. S3). Importantly, modulation of these enzymes leading to the increased local availability of progesterone as a substrate for T production by adjacent HSD17B3-expressing Leydig cells may in part explain why T levels both within the testis and in the circulation are unchanged relative to controls, without modulation of circulating LH levels. A similar paradigm of intercellular steroidogenesis is apparent in the fetal testis, where steroid precursors produced by the fetal Leydig cells undergo final conversion to T by HSD17B3 within the Sertoli cells (66, 67).

In addition to transcriptional changes, Leydig cells lacking AR retain the hyperplastic phenotype seen in the *Tfm*, indicative of an arrest at the immature adult Leydig cell stage. This arrest is similar to that observed in Leydig cells in a previous PTM ARKO (68). Recent data demonstrating that Leydig cells can develop from ACTA2-expressing PTM cells (69) perhaps explain this observation, identifying PTM cells as a potential source of Leydig stem cells in the adult testis. In conclusion, Leydig cell maturation and steroidogenic enzyme expression would appear to be key roles of autocrine AR signaling within Leydig cells because adjacent cells retaining AR expression mature normally (determined both by histology and maturation marker expression, *e.g.*, INSL3) but cannot promote maturation in adjacent AR-negative Leydig cells.

### Autocrine AR signaling in Leydig cells protects against late-onset disruption to spermatogenesis

As discussed above, despite the failure of the majority of Leydig cells to fully mature and the changes in steroidogenic enzyme transcription, circulating plasma T, ITT, and LH levels are all unchanged in LCARKO mice. This is in contrast to the *Tfm* in which serum T levels are significantly decreased despite high levels of LH (20, 21). In line with this normal hormonal profile, spermatogenesis proceeds normally, and LCARKO mice are fertile at d 80. However, from d 80 onward, the LCARKO exhibits a phenotype of focal germ cell loss that becomes increasingly more widespread with age. This could in part be due to a paracrine effect of Leydig cells on PTM cells because DES protein localization is disrupted in the LCARKO testis. However, this influence is likely to be minimal because other PTM markers are normal, and spermatogenesis develops normally, in contrast to the PTM-ARKO mouse, which shows defects in spermatogenesis from d 15 (30).

The Leydig cells make 2 major products: INSL3 and T. INSL3 is almost absent at the transcript level and absent from a majority of Leydig cells at the protein level in the LCARKO. There is some evidence that INSL3 protects against germ cell loss/apoptosis (54, 70, 71); however, recent data from a germ cell-specific KO of *Rxfp2* showed that INSL3 signaling is dispensable for spermatogenesis (55). In contrast, T replacement is able to completely rescue spermatogenesis following EDS-mediated Leydig cell ablation in rats (53). This suggests that the only product produced by Leydig cells, necessary and sufficient for spermatogenesis, is T. However, the LCARKO testis exhibits germ cell loss despite normal ITT levels. The only major difference between the EDS rat model and the LCARKO is that Leydig cells are retained in the LCARKO model, suggesting that the loss of Leydig cell AR results in production of a factor(s) toxic to spermatogenesis. One candidate for this factor would be progesterone, which is significantly increased in the LCARKO testis. However, we were unable to detect any gene expression of *Pgr* in the testis at d 80, suggesting that direct action by progesterone does not underpin this phenotype.

### Autocrine AR signaling in Leydig cells protects against Leydig cell apoptosis

In addition to impacting spermatogenesis, the absence of autocrine AR signaling is associated with premature Leydig cell apoptosis, whereas adjacent AR-positive Leydig cells remain unaffected. Because the onset of Leydig cell apoptosis occurs after the onset of germ cell loss, it is likely that both are separate manifestations of the same underlying dysregulation of Leydig cells in the LCARKO model, possibly acting *via* different pathways, rather than the germ cell loss being somehow directly linked to Leydig cell apoptosis. We cannot rule out the possibility that germ cell loss induces Leydig cell apoptosis, although this appears unlikely because this is not seen in models of germ cell ablation using either busulphan (72, 73) or diphtheria toxin (61), and also would not explain why LC apoptosis is restricted to LCs that lack endogenous AR expression.

Leydig cell apoptosis is not normally observed in the mouse (56), and in the context of the data discussed above, the most likely scenario in LCARKO mice is that the loss of autocrine AR signaling results in production of something toxic to Leydig cell survival. Oxidative damage is associated with Leydig cell apoptosis *in vitro* (74), and it would appear possible that failure to complete maturation would lead to aberrant steroidogenesis, possibly increasing free radical damage. However, acute hCG-mediated stimulation of maximal T output identified no significant difference in T production capacity between LCARKO and control testes at either d 80 or ∼8 mo of age. Because current hypotheses suggest that oxidative stress damage results in a decline in T production (75), this implies that, despite the attractiveness of this argument, oxidative stress is not a major factor in driving increased Leydig cell apoptosis.

### Loss of Leydig cell AR phenocopies models of increased estrogen signaling

Androgen/estrogen balance is important for health, and there is evidence that it is involved in the etiology of obesity (76), cardiovascular disease (77), and benign prostatic hyperplasia (76, 78) as well as infertility. The phenotype of germ cell loss and Leydig cell apoptosis associated with interstitial macrophage phagocytosis observed in the LCARKO testis is similar to 2 mouse models in the literature that have an excess of estrogen signaling in the testis. One is a model with an overexpression of CYP19A1 (Arom), the enzyme responsible for converting androgens into estrogens (58); the other is a global genetic KO of SULT1E1, an enzyme that inactivates estrogens by sulfation (59). We observed no increase in CYP19A1 at either the transcript or protein level in the LCARKO testis, and concentrations of plasma and intratesticular estradiol were normal in the LCARKO model, demonstrating that, although the phenotypes are similar between the Arom+ and LCARKO models, the underlying mechanism differs because AR does not control CYP19A1 expression in Leydig cells. *Sult1e1* shows discrete and highly regulated expression restricted to the placenta, liver, epididymis, and testicular Leydig cells (79–81) and is positively transcriptionally regulated by androgens (82). In contrast to this previous study, we observed a small increase in *Sult1e1* transcript in the LCARKO testis, although the lack of availability of a functioning antibody prevented quantification or spatial localization at the protein level. As such, we cannot rule out the possibly that this small increase in detectable *Sult1e1* transcript arises from Leydig cells that retain AR expression. However, the significant increase in seminal vesicle weight observed in the LCARKO mouse is also seen in the SULT1E1-KO mouse, where it is attributed to increased availability of nonsulfated estrogen, suggesting that modulation of SULT1E1 function in Leydig cells could play a role in the observed LCARKO phenotype; this requires further investigation. Additional evidence supporting increased local estrogen signaling is provided by the observation of an up-regulation in estrogen signaling pathways through ESR1: *Esr1* is up-regulated at the transcript level, and the 46 kDa isoform of ESR1 (83) is significantly increased at the protein level, whereas *Gas6* [an established downstream ESR1 signaling target within Leydig cells (60)] also shows significantly increased transcription. Similar up-regulation of ESR1 is seen in AR-ablated cells in other conditional ARKO models (27, 84, 85), suggesting that AR may act to repress aberrant expression of ESR1 in some cell types, probably *via* an intermediary factor. Finally, consistent with this conclusion, patients with CAIS show a well-documented significant increase in estrogen production at puberty (86), which closely follows the ontogeny of Leydig cell apoptosis in CAIS testes observed in this study. This suggests that the loss of Leydig cell AR signaling results in failure to oppose local estrogen signaling, leading to late-onset testicular degeneration and Leydig cell apoptosis in both mice and humans.

Although a model of increased estrogen signaling in the LCARKO is extremely attractive, especially in light of a recently published follow-up study investigating the mechanism of Leydig cell apoptosis in the Arom+ mouse, which shares many of the features we observe in the LCARKO (60), we are unable to rule out the possibility that the similar phenotypes of the Arom+, SULT1E1-KO, and the LCARKO models all reflect a general response to perturbation of Leydig cell maturation and adult Leydig cell function. It is clear that elucidating these mechanisms requires significant further investigation. Furthermore, whereas we clearly demonstrate several key roles for autocrine androgen action with Leydig cells, we cannot rule out additional roles for Leydig cell AR signaling that may be revealed through ablation of AR action from all Leydig cells.

Nevertheless, in conclusion, these analyses identify important and hitherto unknown autocrine roles for androgen signaling in Leydig cell maturation and testis function. Our identification that perturbed androgen action in the developing Leydig cell lineage leads to a failure of Leydig cell maturation and perturbed function, associated with late-onset degeneration of spermatogenesis in mice and Leydig cell apoptosis in both mice and humans, is an important finding that may have implications for men during aging.

## Supplementary Material

Supplemental Data

## Abbreviations

Adipo-ARKO: adipocyte androgen receptor knockout
AR: androgen receptor
ARKO: androgen receptor knockout
Arom: aromatase
CAIS: complete androgen insensitivity syndrome
CASP3: caspase-3
DAB: 3,3′-diaminobenzidine
DES: desmin
EDS: ethane dimethane sulfonate
*Fabp4*: fatty acid binding protein 4
FSH: follicle-stimulating hormone
GAPDH: glyceraldehyde 3-phosphate dehydrogenase
hCG: human chorionic gonadotropin
HSD3B: 3β-hydroxysteroid dehydrogenase
ITT: intratesticular testosterone
KO: knockout
LCARKO: Leydig cell androgen receptor knockout
PTM: peritubular myoid
T: testosterone
*Tfm*: testicular feminization mouse
YFP: yellow fluorescent protein
