# Supplementary material for: Autocrine androgen action is essential for Leydig cell maturation and function, and protects against late-onset Leydig cell apoptosis in both mice and men
Source: FASEB J. 2014 Nov 17;29(3):894–910. doi: 10.1096/fj.14-255729 (PMC4422361; doi:10.1096/fj.14-255729)
Supplement: Supplemental Data [file supp_29_3_894__index.html]

Autocrine androgen action is essential for Leydig cell maturation and function, and protects against late-onset Leydig cell apoptosis in both mice and men — Supplemental Data 

# Autocrine androgen action is essential for Leydig cell maturation and function, and protects against late-onset Leydig cell apoptosis in both mice and men

## Supplemental Data

**Files in this Data Supplement:**

- Supplemental Data
- Supplemental Data
- Supplemental Data
- Supplemental Data
