# Supplementary material for: Autocrine androgen action is essential for Leydig cell maturation and function, and protects against late-onset Leydig cell apoptosis in both mice and men
Source: FASEB J. 2014 Nov 17;29(3):894–910. doi: 10.1096/fj.14-255729 (PMC4422361; doi:10.1096/fj.14-255729)
Supplement: Supplemental Data [file supp_fj.14-255729_Supplemental_Figure1.pdf]

SUPPLEMENTARY FIGURE 1

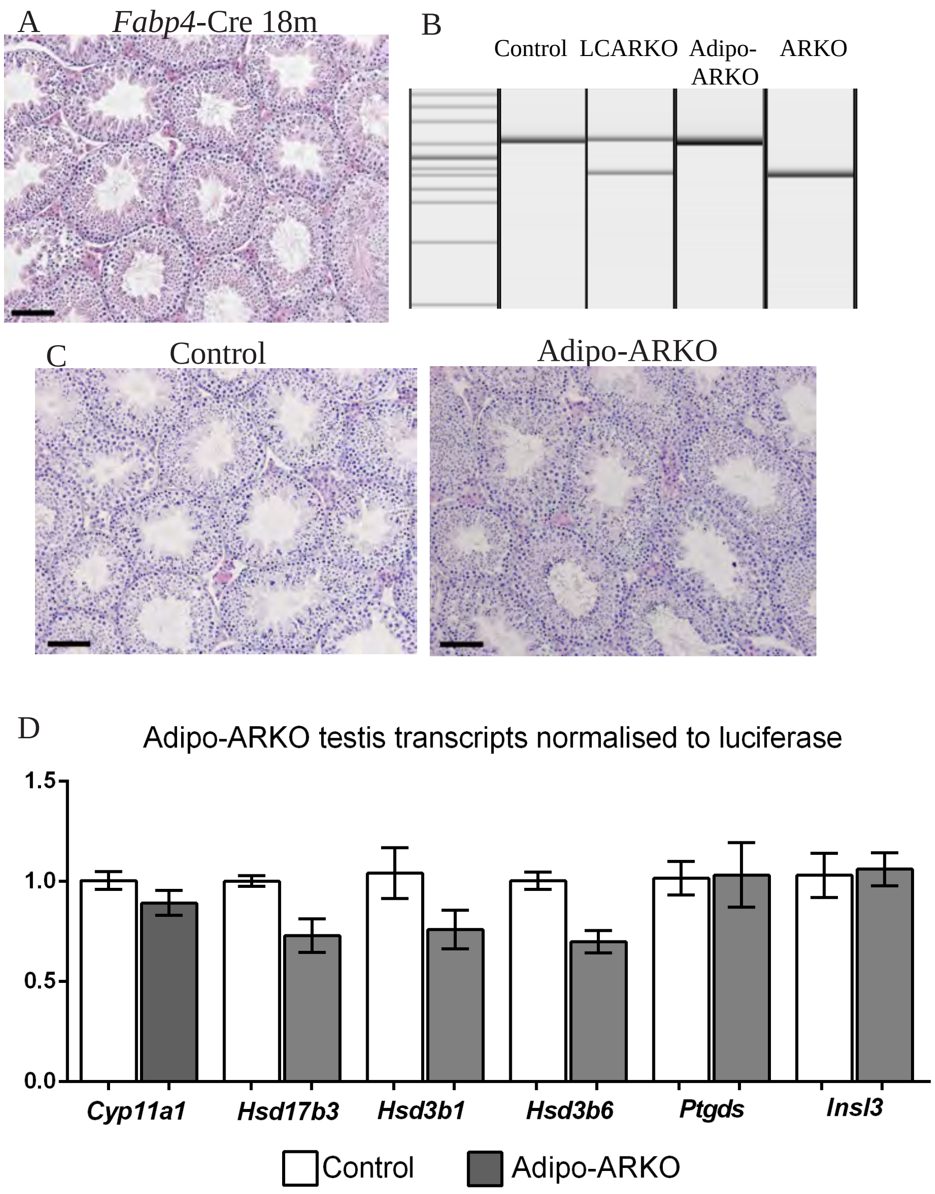

Supplementary Figure 1

Scale bars 100 µm

A. Fabp4-Cre testis histology is normal at 18 months of age.

B. Genomic AR in control and Adipo-ARKO testis is not recombined (1142 bp band). Genomic recombination of AR (indicated by a 612 bp band) occurs in a population of the cells of the LCARKO testis, another population has unrecombined AR. All genomic AR in ARKO testes is recombined.

C. Adipo-ARKO testis histology is similar to controls.

D. There are no change in steroidogenic and Leydig cell maturation markers in the Adipo-ARKO testis compared to controls (n=6 for each group).
