# Supplementary material for: Autocrine androgen action is essential for Leydig cell maturation and function, and protects against late-onset Leydig cell apoptosis in both mice and men
Source: FASEB J. 2014 Nov 17;29(3):894–910. doi: 10.1096/fj.14-255729 (PMC4422361; doi:10.1096/fj.14-255729)
Supplement: Supplemental Data [file supp_fj.14-255729_Supplemental_Figure2.pdf]

SUPPLEMENTARY FIGURE 2

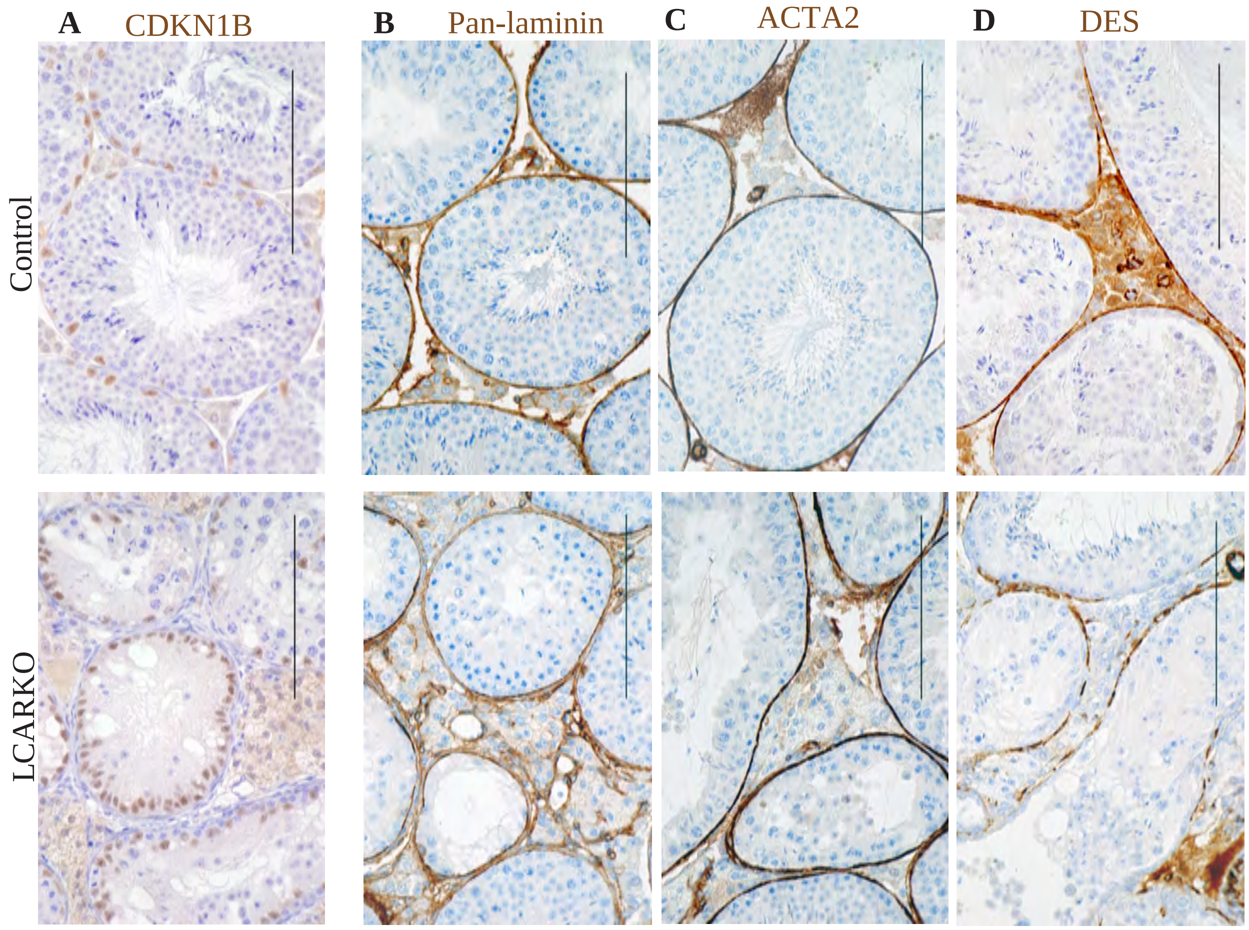

Supplementary Figure 2

Scale bars are 100 µm

A. Anti-CDKN1B stains Sertoli cells in both control and LCARKO testes at d80.

B. Anti-pan Laminin has an unbroken peritubular staining pattern in both control and LCARKO testes at d80.

C. Anti-ACTA2 has an unbroken peritubular staining pattern in both control and LCARKO testes at d80.

D. Anti-DES has an unbroken peritubular staining pattern in d80 control testes, but staining is interrupted in d80 LCARKO testes.
