# Supplementary material for: Autocrine androgen action is essential for Leydig cell maturation and function, and protects against late-onset Leydig cell apoptosis in both mice and men
Source: FASEB J. 2014 Nov 17;29(3):894–910. doi: 10.1096/fj.14-255729 (PMC4422361; doi:10.1096/fj.14-255729)
Supplement: Supplemental Data [file supp_fj.14-255729_Supplemental_Figure3.pdf]

SUPPLEMENTARY FIGURE 3

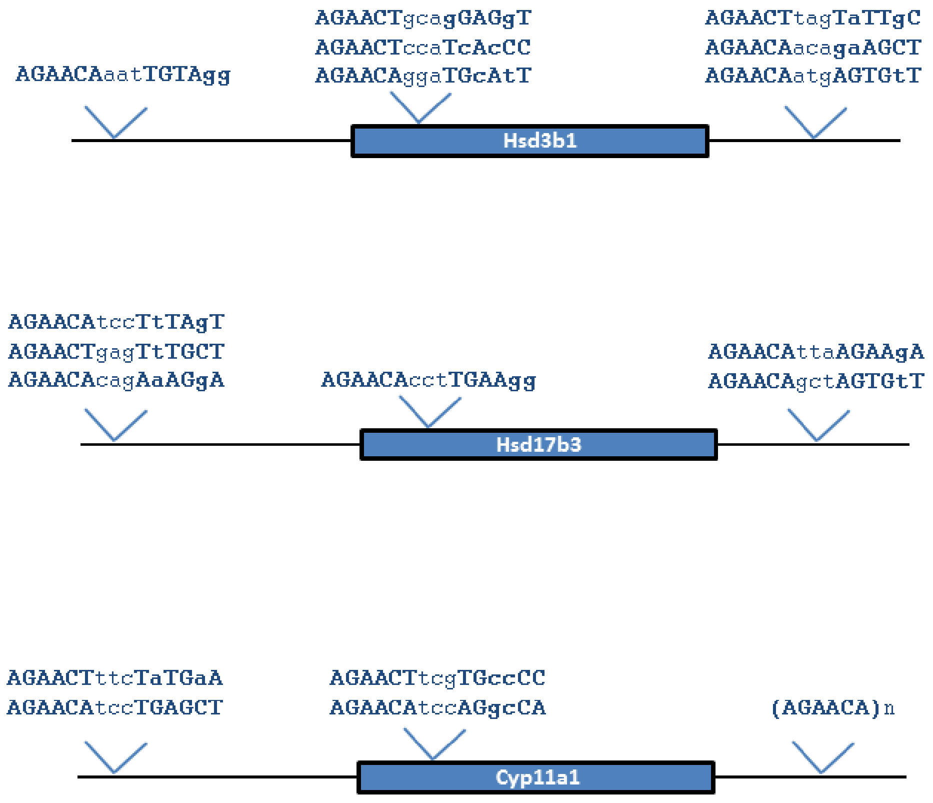


**Supplementary Figure 3**

Locations and sequences of putative AREs in the Hsd3b1, Hsd17b3 and Cyp11a1 genes
