# Supplementary material for: Autocrine androgen action is essential for Leydig cell maturation and function, and protects against late-onset Leydig cell apoptosis in both mice and men
Source: FASEB J. 2014 Nov 17;29(3):894–910. doi: 10.1096/fj.14-255729 (PMC4422361; doi:10.1096/fj.14-255729)
Supplement: Supplemental Data [file supp_fj.14-255729_Supplemental_Table1.pdf]

Supplementary Table 1: Cre Recombinase Line Characterisation

Cre line	Testis distribution in our hands	Our Publication(s) using this Cre line
Amh-Cre	Sertoli cells	Rebourcet D, et al; (2014) *PLoS ONE* 9(8):<br><br>Rebourcet D, et al; (2014) *Development* 141:2139-2149
smMHC-Cre	Peritubular Myoid cells/arteriole smooth muscle	Welsh M, et al; (2009) *FASEB J.* 23:4218-30<br><br>Welsh M, et al; (2012) *International Journal of Andrology* 35: 25-40
SM22-Cre	Arteriole smooth muscle	Welsh M, et al; (2010) *PLoS One* 5(10): e13632.
Tie2-Cre	Vascular endothelial cells	O'Hara L and Smith LB. (2012) BMC Research Notes 5:16 doi:10.1186/1756-0500-5-16
Aquaporin2-Cre	spermatids	Smith LB (2011) Reproduction. 141(2):151-61
Stra8-Cre	prespermatogonia	Rebourcet D, et al; (2014) *PLoS ONE* 9(8):<br><br>Rebourcet D, et al; (2014) *Development* 141:2139-2149
Cyp11a1-Cre	Leydig cells	O'Hara L, et al; (2014) PLoS ONE 9(1): e84541. doi:10.1371/journal.pone.0084541
